# Smad2/3 Upregulates the Expression of Vimentin and Affects Its Distribution in DBP-Exposed Sertoli Cells

**DOI:** 10.1155/2015/489314

**Published:** 2015-12-24

**Authors:** Xi Zhang, Xiaogang Wang, Taixiu Liu, Min Mo, Lin Ao, Jinyi Liu, Jia Cao, Zhihong Cui

**Affiliations:** Institute of Toxicology, College of Preventive Medicine, Third Military Medical University, No. 30, Gaotanyan Road, Shapingba District, Chongqing 400038, China

## Abstract

Sertoli cells (SCs) in the testes provide physical and nutritional support to germ cells. The vimentin cytoskeleton in SCs is disrupted by dibutyl phthalate (DBP), which leads to SCs dysfunction. In a previous study, we found that peroxisome proliferator-activated receptor alpha (PPAR*α*) influenced the distribution of vimentin by affecting its phosphorylation in DBP-exposed SCs. In the present study, we investigated the role of Smad2/3 in regulating the expression of vimentin in DBP-exposed SCs. We hypothesized that Smad2/3 affects the distribution of vimentin by regulating its expression and that there is cross talk between Smad2/3 and PPAR*α*. The real-time PCR and ChIP-qPCR results showed that SB431542 (an inhibitor of Smad2/3) could significantly attenuate the expression of vimentin induced by DBP in SCs. Phosphorylated and soluble vimentin were both downregulated by SB431542 pretreatment. WY14643 (an agonist of PPAR*α*) pretreatment stimulated, while GW6471 (an antagonist of PPAR*α*) inhibited, the activity of Smad2/3; SB431542 pretreatment also inhibited the activity of PPAR*α*, but it did not rescue the DBP-induced collapse in vimentin. Our results suggest that, in addition to promoting the phosphorylation of vimentin, DBP also stimulates the expression of vimentin by activating Smad2/3 in SCs and thereby induces irregular vimentin distribution.

## 1. Introduction

Spermatogenesis is an essential process for male reproductive function. In one report, subfertility or infertility was generally due to defects in spermatogenesis [1]. Dibutyl phthalate (DBP) is a plasticizer that is widely distributed in the environment and frequently contacted by human. Previous epidemiological, animal, and molecular studies have shown that DBP disrupted spermatogenesis [2–6]. Spermatogenesis occurs in the seminiferous tubules; SCs are unique somatic cells in the seminiferous tubules, which provide physical and nutritional support for spermatocyte maturation [7, 8]. Periodically “open” and “closed” junctions between SCs and germ cells mediate the maturation process of spermatocyte [9]. Vimentin is tightly connected to the desmosome structure linking germ cells and SCs. The distribution of vimentin filaments changes dynamically during the seminiferous epithelium cycle [10]. Previous studies have shown that the vimentin cytoskeleton was disrupted in DBP-exposed SCs [11]. However, the mechanism of this process is still unknown.

In our previous study, we found that levels of phosphorylated and soluble vimentin were higher in DBP-exposed SCs compared to GW6471 + DBP-treated SCs [12]. We did not investigate vimentin mRNA expression, but the expression of vimentin may also affect the concentration of soluble vimentin. According to a previous study, vimentin mRNA was upregulated in DBP-exposed SCs [13].

The expression of vimentin mRNA was regulated by Smad2/3 in several other cell lines, such as esophageal squamous cell carcinoma cell lines (e.g., Eca109, EC9706, and KYSE150), squamous cell carcinoma of the head and neck cell line Tu686, skeletal myogenic cell line C2C12, and normal human epidermal keratinocytes cells. Smad2/3 binds to the Smad binding element in the promoter region of vimentin to regulate its expression [14–17]. In this study, we investigated the role of Smad2/3 in the regulation of vimentin mRNA expression in DBP-exposed SCs.

We hypothesized that Smad2/3 could affect the distribution of vimentin by regulating its mRNA expression in DBP-exposed SCs, possibly through cross talk with PPAR*α*; these two factors cooperate to affect the distribution of vimentin. Chromatin immunoprecipitation and quantitative real-time PCR (ChIP-qPCR) analysis was performed to identify the regulatory region in the promoter of vimentin bound by phosphorylated Smad2/3. The distribution of vimentin in SCs after different treatments was determined with immunocytochemistry and immunofluorescence, and treatments with various agonists or antagonists were used to investigate the relationship between Smad2/3 and PPAR*α*.

## 2. Materials and Methods

### 2.1. Animal and Reagents

Three-week-old male Sprague-Dawley (SD) rats were purchased from the Center of Experimental Animals at Third Military Medical University (Chongqing, China). They were used under the 3R (replacement, reduction, and refinement) principles. DBP and 4′,6-diamidino-2-phenylindole (DAPI) were purchased from Sigma-Aldrich Co. (St. Louis, MO, USA). WY14643 (sc-203314), GW6471 (sc-300779), SB431542 (sc-204265), antibodies for vimentin (V9, sc-6260), p-vimentin (Ser83, sc-16674-R), PPAR*α* (N-19, sc-1985), p-Smad2/3 (sc-11769), and Smad2/3 (sc-8332) were purchased from Santa Cruz Biotechnology (Santa Cruz, CA, USA). An antibody for *β*-actin (AA128), HRP-labeled secondary antibodies (A0216, A0208, and A0181), western/IP cell lysis buffer (P0013), a BCA protein assay kit (P0010), an enhanced chemiluminescent kit (P0018A), a ChIP assay kit (P2078), and a PCR/DNA purification kit (D0033) were purchased from Beyotime Institute of Biotechnology (Beyotime Institute of Biotechnology, Haimen, China). DyLight 488 AffiniPure Goat Anti-Mouse IgG (H + L) was purchased from Earth Ox (San Francisco, CA, USA). 0.22 *μ*m PVDF membrane, 0.45 *μ*m NC membranes, and the enhanced chemiluminescent kit were purchased from Millipore Corporation (Billerica, MA, USA). The 3,3′-diaminobenzidine (DAB) kit used in immunocytochemical experiments was purchased from Zhongshan Golden Bridge Biotechnology (ZSBiO, Beijing, China). Primers were synthesized by Shanghai Sangon Biological Engineering Technology & Services Co. (Shanghai, China). The reverse transcription kit (RR047A) was purchased from TaKaRa Biotechnology Co. (Dalian). The quantitative real-time PCR kit (A6001) was purchased from Promega Co., an affiliate of Promega (Beijing) Biotech Co., Ltd.

### 2.2. Sertoli Cell Preparation

Primary SCs were isolated according to previously described procedures [18–21]. In brief, trypsin (HyClone, Rockford, IL, USA) and collagenase IV (Sigma-Aldrich, St. Louis, MO, USA) were used to digest the scissored testes tissue blocks for 30–60 min at 37°C. After stopping the digestion by addition of 10% FBS (Gibco, USA) containing Dulbecco's modified Eagle's medium/Ham's nutrient mixture F12 (DMEM/F12, 1 : 1) medium (pH 7.2) (HyClone, Thermo Fisher Scientific, Rockford, IL, USA), the mixture was sieved through sterilized 100 meshes. Newly separated cells were cultured for 48 hours in 10 cm diameter dishes (Corning, New York, USA) with DMEM/F12, 10% FBS, and 1x penicillin-streptomycin (Beyotime Institute of Biotechnology, Haimen, China) in 5% CO_2_ at 35°C. Then, cells were treated with 20 mM Tris-HCl (pH 7.4) for 2.5 min.

### 2.3. Cell Culture and Treatments

Approximately 2 × 10^6^ SCs were seeded into 10 cm dishes. DBP was diluted in DMSO (dimethyl sulfoxide) to 100 mM. Ten microliters of DBP was added to each 10 cm dish, for a final concentration of 100 *μ*M. Equal volumes of DMSO were added to control dishes. Agonists or antagonists (GW6471, WY14643, and SB431542) were added two hours before DBP treatment at a concentration of 10 *μ*M, according to previous studies [14, 22, 23]. SCs were treated with DMSO, DBP, SB431542 + DBP, and SB431542 for 24 h to extract RNA. SCs were treated with DBP, WY14643 + DBP, GW6471 + DBP, or SB431542 + DBP for 0 h, 1 h, 3 h, 6 h, 12 h, and 24 h, respectively, to extract protein. The dosage of DBP was based on the CCK8 and immunofluorescence experiments. In the CCK8 experiment, we added 0, 20, 40, 60, 80, 100, and 200 *μ*M of DBP to detect its toxicity in SCs. The results showed that DBP promoted Sertoli cell proliferation in a dose-dependent manner. We selected 100 *μ*M because its adverse effect on vimentin in SCs at the 24 h time point was apparent but not severe enough to interfere with western blotting. We selected a series of time points within 24 h because the damage induced by DBP to SCs was too severe at 48 h or 72 h. Moreover, we also took into account the dosage and time points used by other researchers [24].

### 2.4. Real-Time PCR

RNA was extracted with TRIzol (Invitrogen, USA) from the treated SCs. Concentrations and purity of RNA were quantified by a Nanodrop 2000 (Thermo Fisher Scientific, Rockford, IL, USA). Approximately 1 *μ*g total RNA was used to generate the cDNA using the PrimeScript RT Reagent Kit with gDNA Eraser (Perfect Real Time). For the PCR reaction, 7 *μ*L nuclease-free water, 10 *μ*L GoTaq Probe qPCR Master Mix (2x), and 1 *μ*L cDNA and primers were used, respectively, in a 20 *μ*L total reaction volume. Real-time PCR was performed using a Bio-Rad IQ5 (Bio-Rad, USA) with a preheated lid up to 105°C, initial denaturation at 95°C for 5 min, and 40 repeats of (95°C for 10 s, 60°C for 30 s), followed by extension at 72°C for 5 min, and the data were analyzed using the 2^(−ΔΔCt)^ method. Primers used in this experiment are listed in Table 1.

### 2.5. ChIP-qPCR

The ChIP assay kit (P2078) used in this experiment was purchased from Beyotime Institute of Biotechnology (Haimen, China). Detailed procedures for ChIP-qPCR were previously described [12]. An anti-p-Smad2/3 antibody was used in this experiment (1 *μ*g). Primers used in this experiment were targeted to Smad binding elements (SBE) in promoter regions of vimentin [25]. We designed four pairs of primers flanking SBE-containing sequences, 2000 bp upstream of the transcription start site of vimentin. Primer sequences are listed in Table 1. After the preliminary experiment, only the primers targeting region −837 to −924 showed high amplification efficiency, so we chose this region for further experiments. The product size was 88 bp.

### 2.6. Western Blotting

Proteins from the SCs were extracted at 0 h, 1 h, 3 h, 6 h, 12 h, and 24 h, respectively, after DBP, GW6471 + DBP, WY14643 + DBP, or SB431542 + DBP treatments. Western/IP cell lysis buffer (20 mM, pH 7.5 Tris, 150 mM NaCl, 1% Triton X-100, sodium pyrophosphate, *β*-glycerophosphate, EDTA, Na_3_VO_4_, and leupeptin) was used as the protein extraction buffer. Protein concentrations were determined by a BCA protein assay kit. Then, 20 to 40 *μ*g sample protein was loaded onto 5%–12% sodium dodecyl sulfate polyacrylamide gels for electrophoresis (SDS-PAGE). Proteins in the gel were either transferred to polyvinylidene fluoride (PVDF) membranes at 18 V for 30 min using a Trans-Blotting SD Semi-Dry Electrophoretic Transfer Cell (Bio-Rad, USA) or transferred to PVDF or nitrocellulose membranes by wet transferring at 80 V or 70 V for 2 h at 4°C or in an ice-water containing container. Transferred membranes were blocked by 5% skim milk powder in TBST for 1 to 2 h at room temperature. First, antibodies were incubated with the membrane at 4°C overnight. The dilution ratio for the actin antibody was 1 : 1000; for other antibodies, it was 1 : 500. After three washes with 0.1% TBST for 5 min each, secondary antibodies were incubated with the membrane at room temperature for 1 h or first incubated at 4°C for 4 h then incubated at room temperature for 30 min or 1 h. Dilution ratios of secondary antibodies were all 1 : 1000. Finally, membranes were washed with 0.1% TBST for three times, 5 min each. The signal was developed using an enhanced chemiluminescent kit purchased from Millipore Corporation (Billerica, MA, USA). FUSION FX5 Spectra (Vilber, France) was used to image the membranes. Densitometric analyses of immunoblots were performed by Image J software. Phosphoprotein and total protein levels were normalized by actin.

### 2.7. Immunofluorescence Assays

Confocal culture dishes (20 mm diameters, 801001, Corning, New York, USA) were used to perform the immunofluorescence experiments. After treatment for 24 h, SCs were fixed in 4% paraformaldehyde (PFA) for 15 min and then treated with 0.1% Triton X-100 (diluted with phosphate-buffered saline). After three washes with PBS, fixed cells were incubated with vimentin antibody (1 : 500) at 4°C overnight. After another three washes with PBS, cells were incubated with DyLight 488 AffiniPure Goat Anti-Mouse IgG (H + L) secondary antibody (1 : 1000) at room temperature for 1 h. Then, the cells were incubated with DAPI for 30 min and washed with PBS three times. Leica TCS-SP5 confocal laser scanning microscope (Leica, Mannheim, Germany) was used to visualize the distribution of vimentin.

### 2.8. Immunocytochemical Assays

SCs were cultured in 48-well plates, and, following the different treatments, cells were fixed in 4% PFA for 15 min. After three washes with PBS, cells were treated with 0.1% Triton X-100 in PBS for 10 min. Then, cells were treated with H_2_O_2_ for 10 min. After three washes with PBS, cells were incubated with 5% bovine serum albumin in PBS at room temperature for 1 h. Then, cells were incubated with vimentin antibody at 4°C overnight. Following three washes with PBS, cells were incubated with horseradish peroxidase- (HRP-) labeled anti-mouse secondary antibody (ZSBiO, Beijing, China) for 1 h at room temperature. After 4 washes, cells were incubated with DAB-chromogen and observed under the microscope until the appropriate brown color appeared. Then, cells were washed thoroughly with PBS. A Nikon Eclipse TE2000 S inverted microscope under 200x original magnification and Nikon digital sight (Nikon, Japan) were used to capture pictures.

### 2.9. Statistical Analysis of Western Blotting, Real-Time PCR, and ChIP-qPCR Results

Differences between groups were evaluated by one-way analysis of variance (ANOVA), followed by the Holm-Sidak test using Sigma Stat 3.5 software (Sigma-Aldrich, St. Louis, MO, USA). *P* < 0.05 was considered to be statistically significant.

## 3. Results

### 3.1. Role of Smad2/3 in Regulating Vimentin mRNA Expression

**Figure 1:**
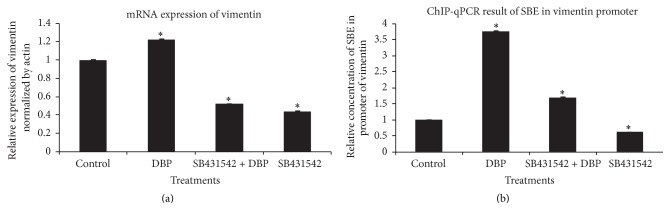
Real-time PCR of vimentin and ChIP-qPCR of Smad binding element (SBE) in the promoter region of vimentin in control and DBP-, SB431542 + DBP-, and SB431542-treated SCs at 24 h. (a) Real-time PCR of vimentin in the indicated SCs. (b) ChIP-qPCR of SBE in the vimentin promoter in the indicated SCs. *P* < 0.05 was considered statistically significant.

Real-time PCR was used to measure vimentin mRNA expression in control and DBP-, Smad2/3 inhibitor- (SB431542-), and DBP + SB431542-treated SCs. DBP-exposed SCs presented significant vimentin mRNA levels increase to 1.22 ± 0.009-fold variation relative to cells cultured in control conditions (Figure 1(a)), and SB431542 + DBP- or SB431542-treated SCs significantly decreased vimentin mRNA levels to 0.52 ± 0.002- or 0.44 ± 0.004-fold variation relative to SCs cultured in control conditions, respectively. We designed four pairs of primers to amplify SBE-containing sequences in the promoter region of vimentin. The most efficient pair of primers was selected to perform the ChIP-qPCR experiment. As showed in Figure 1(b), binding of p-Smad2/3 to the SBE of vimentin in DBP-treated SCs significantly increased to 3.77 ± 0.009-fold variation relative to SCs cultured in control conditions, and the inhibition of Smad2/3 by SB431542 could significantly decrease the binding of p-Smad2/3 to the SBE of vimentin to 1.70 ± 0.01-fold variation in SB431542 + DBP-treated SCs and to 0.63 ± 0.003-fold variation in SB431542-treated SCs relative to SCs cultured in control conditions. ChIP-qPCR results were consistent with the real-time PCR results. In summary, these findings indicate that Smad2/3 upregulated vimentin mRNA expression in DBP-exposed SCs.

### 3.2. Role of Smad2/3 in Regulating Vimentin Protein Expression

**Figure 2:**
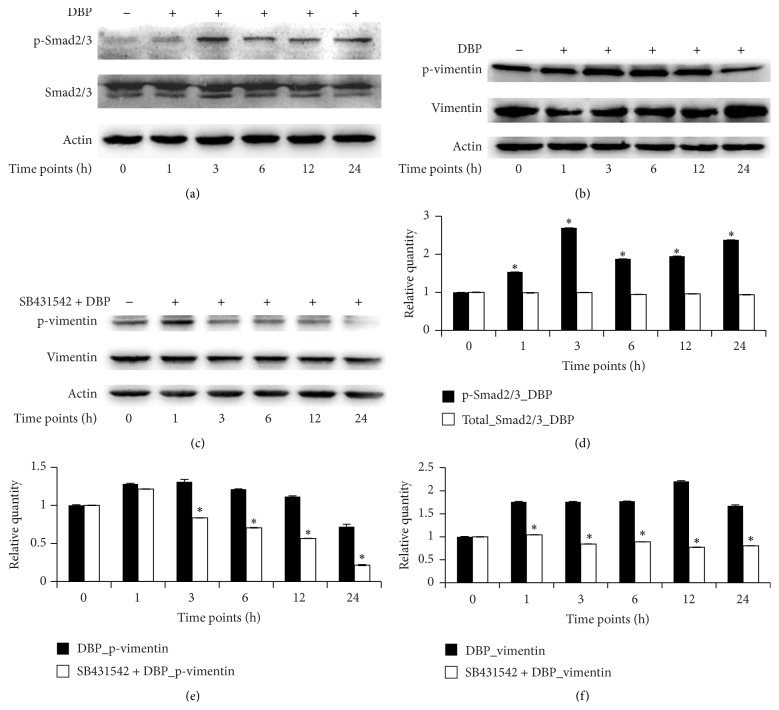
Effects of Smad2/3 on phosphorylation and expression of vimentin in DBP-treated SCs at 0, 1, 3, 6, 12, and 24 h. (a), (b), and (c) show western blotting results of p-Smad2/3, Smad2/3, p-vimentin, vimentin, and actin in DBP- or SB431542 + DBP-treated SCs at different time points. (d), (e), and (f) show the relative quantitative analysis of western blotting results by Image J software. *P* < 0.05 was considered statistically significant.

Western blotting was used to detect the activity of p-Smad2/3 in DBP-treated SCs, and phosphorylated and soluble vimentin were detected in the DBP- and SB431542 + DBP-treated SCs. As showed in Figures 2(a) and 2(d), relative concentrations of total Smad2/3 protein compared to actin remained stable within 24 h in DBP-treated SCs; relative concentration of p-Smad2/3 compared to actin in DBP-treated SCs was significantly increased to 1.53 ± 0.002-fold variation at 1 h, 2.69 ± 0.0005-fold variation at 3 h, 1.87 ± 0.0009-fold variation at 6 h, 1.94 ± 0.006-fold variation at 12 h, and 2.37 ± 0.0002-fold variation at 24 h relative to SCs cultured in control conditions, respectively. To determine the effect of Smad2/3 on phosphorylated and soluble vimentin, western blotting was performed. As showed in Figures 2(b) and 2(e), phosphorylated vimentin levels in DBP-treated SCs increased to 1.28 ± 0.009-fold variation at 1 h, to 1.30 ± 0.03-fold variation at 3 h, to 1.21 ± 0.003-fold variation at 6 h, and to 1.11 ± 0.01-fold variation at 12 h and then decreased to 0.71 ± 0.03-fold variation at 24 h relative to SCs at 0 h. As showed in Figures 2(c) and 2(e), phosphorylated vimentin levels increased in SB431542 + DBP-treated cells to 1.21 ± 0.002-fold variation at 1 h and then decreased to 0.83 ± 0.001-fold variation at 3 h, to 0.70 ± 0.004-fold variation at 6 h, to 0.56 ± 0.007-fold variation at 12 h, and to 0.21 ± 0.005-fold variation at 24 h relative to SCs at 0 h. Phosphorylated vimentin levels were significantly lower at each time point in SB431542 + DBP-treated than DBP-treated cells (*P* < 0.05). The relative quantity of soluble vimentin to actin in DBP-treated SCs increased to 1.76 ± 0.006-fold variation at 1 h, to 1.75 ± 0.008-fold variation at 3 h, to 1.77 ± 0.004-fold variation at 6 h, to 2.20 ± 0.01-fold variation at 12 h, and to 1.67 ± 0.02-fold variation at 24 h relative to SCs at 0 h. Soluble vimentin in SB431542 + DBP-treated SCs decreased to 1.04 ± 0.003-fold variation at 1 h, to 0.84 ± 0.002-fold variation at 3 h, to 0.89 ± 0.001-fold variation at 6 h, to 0.77 ± 0.002-fold variation at 12 h, and to 0.80 ± 0.001-fold variation at 24 h, relative to SCs at 0 h. Soluble vimentin levels were significantly higher in the DBP-treated SCs than the SB431542 + DBP-treated cells at each time point (*P* < 0.05). In summary, Smad2/3 affected the phosphorylation and solubility of vimentin in DBP-exposed SCs.

### 3.3. Relationship between PPAR*α* and Smad2/3 in DBP-Treated SCs

**Figure 3:**
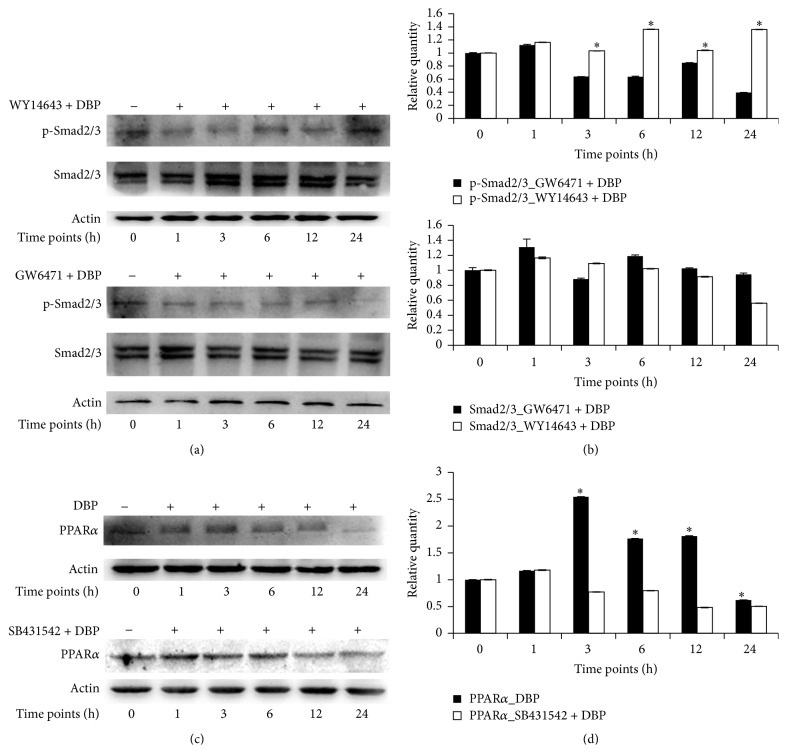
Relationship between PPAR*α* and Smad2/3 in DBP-treated SCs. GW6471, WY14643, and SB431542 were added to the cultural medium 2 h before addition of DBP, respectively. Western blotting was used to test levels of p-Smad2/3 and Smad2/3 in WY14643 + DBP- and GW6471 + DBP-treated SCs (as shown in (a)) and levels of PPAR*α* in DBP- and SB431542 + DBP-treated SCs, respectively (as shown in (c)). (b) and (d) show the relative quantitative analysis of parts (a) and (c), respectively. *P* < 0.05 was considered statistically significant.

An antagonist (GW6471) and an agonist (WY14643) of PPAR*α* were used to investigate the effect of PPAR*α* on the activity of Smad2/3. As shown in Figures 3(a) and 3(b), the relative quantity of p-Smad2/3 to actin in WY14643 + DBP-treated SCs increased to 1.16 ± 0.003-fold variation at 1 h, to 1.03 ± 0.008-fold variation at 3 h, to 1.36 ± 0.003-fold variation at 6 h, to 1.04 ± 0.004-fold variation at 12 h, and to 1.35 ± 0.002-fold variation at 24 h relative to SCs at 0 h. The relative quantity of p-Smad2/3 to actin in GW6471 + DBP-treated SCs increased to 1.11 ± 0.01-fold variation at 1 h and then decreased to 0.63 ± 0.004-fold variation at 3 h, to 0.63 ± 0.007-fold variation at 6 h, to 0.85 ± 0.003-fold variation at 12 h, and to 0.39 ± 0.004-fold variation at 24 h relative to SCs at 0 h. The relative quantity of p-Smad2/3 was significantly higher in WY14643 + DBP-treated SCs than GW6471 + DBP-treated cells at each time point (*P* < 0.05). Smad2/3 levels in the WY14643 + DBP-treated SCs increased to 1.30 ± 0.1-fold variation at 1 h, to 1.09 ± 0.006-fold variation at 3 h, and to 1.02 ± 0.003-fold variation at 6 h and decreased to 0.91 ± 0.004-fold variation at 12 h and to 0.56 ± 0.003-fold variation at 24 h relative to SCs at 0 h. Smad2/3 levels in the GW6471 + DBP-treated SCs increased to 1.16 ± 0.01-fold variation at 1 h, to 1.18 ± 0.01-fold variation at 6 h, and to 1.02 ± 0.008-fold variation at 12 h and decreased to 0.88 ± 0.01-fold variation at 3 h and to 0.94 ± 0.02-fold variation at 24 h relative to SCs at 0 h. There were no significant differences in levels of Smad2/3 between WY14643 + DBP-treated and GW6471 + DBP-treated SCs at 1 h, 6 h, and 12 h (*P* < 0.05). The level of Smad2/3 was significantly (*P* < 0.05) higher in WY14643 + DBP-treated than GW6471 + DBP-treated SCs at 3 h and less at 24 h.

In addition, an inhibitor of p-Smad2/3 (SB431542) was used to assess the influence of Smad2/3 on the activity of PPAR*α*. As shown in Figures 3(c) and 3(d), PPAR*α* levels in DBP-treated SCs increased to 1.16 ± 0.002-fold variation at 1 h, to 2.54 ± 0.002-fold variation at 3 h, to 1.76 ± 0.003-fold variation at 6 h, and to 1.81 ± 0.005-fold variation at 12 h and then decreased to 0.62 ± 0.001-fold variation at 24 h relative to SCs at 0 h; the relative quantity of PPAR*α* in SB431542 + DBP-treated cells increased to 1.17 ± 0.006-fold variation at 1 h and then decreased to 0.77 ± 0.003-fold variation at 3 h, to 0.79 ± 0.004-fold variation at 6 h, to 0.48 ± 0.004-fold variation at 12 h, and to 0.50 ± 0.002-fold variation at 24 h relative to SCs at 0 h. PPAR*α* levels were significantly (*P* < 0.05) higher in DBP-treated SCs than SB431542 + DBP-treated cells at 3 h, 6 h, 12 h, and 24 h. This result suggested that DBP could activate PPAR*α*, and the activation of PPAR*α* in turn activated p-Smad2/3, initiating a positive feedback loop with PPAR*α*. In summary, WY14643 induced the activity of Smad2/3, GW6471 inhibited the activity of Smad2/3, and SB431542 inhibited the activity of PPAR*α* in DBP-exposed SCs.

### 3.4. Distribution of Vimentin in DBP- and SB431542 + DBP-Treated SCs at 24 h

**Figure 4:**
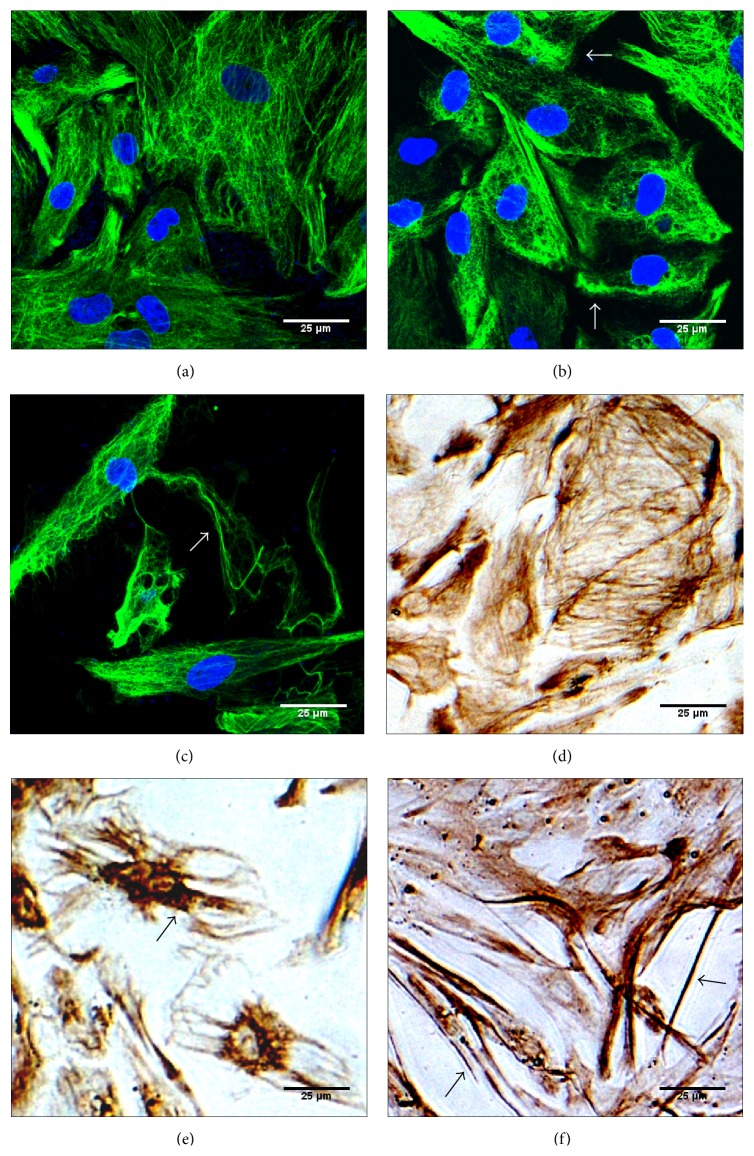
Immunofluorescence and immunocytochemical experiments of vimentin in control and DBP- and SB431542 + DBP-treated SCs at 24 h. (a) and (d) represent the control group; (b) and (e) represent the DBP-treated group; (c) and (f) represent the SB431542 + DBP-treated group (magnification: 200x). The distribution of vimentin showed abnormal aggregation in subregions of SCs (as shown by arrows) compared with the control group. The distribution of vimentin in the SB431542 + DBP-treated group showed many irregular long filamentous structures.

Immunofluorescence and immunocytochemical experiments were performed to detect the distribution of vimentin in control and DBP- and DBP + SB431542-treated cells at 24 h. As shown in Figures 4(a) and 4(d), vimentin was distributed regularly in the control group and was mainly present as a network structure, with abnormal aggregated vimentin seldom observed. As shown in Figures 4(b) and 4(e), regular distribution of vimentin was disrupted in DBP-treated cells, abnormal aggregated vimentin was partially distributed to regions of the cell, and a large proportion of the cells showed concentrated vimentin around the nucleus. As shown in Figures 4(c) and 4(f), vimentin in SB431542 + DBP-treated SCs was abundantly present as irregular long filamentous structures which differed from the DBP-treated cells. In summary, SB431542 did not reverse the collapse of vimentin induced by DBP but triggered the distribution of vimentin as irregular long filaments.

## 4. Discussion

Spermatogenesis occurs in the seminiferous tubules, and SCs are unique somatic cells in the seminiferous tubules [26] that provide structural and nutritional support during the process of germ cell maturation [27]. Vimentin is an important component of the desmosome [28], and disruption of the vimentin cytoskeleton by DBP leads to dysfunction of the desmosomes [24]. Without the structural support and nutritional supply from SCs, germ cells are prone to apoptosis [29].

Our previous results demonstrated that the increased phosphorylation of vimentin by PPAR*α* was the main mechanism of the disruption of vimentin in SCs [12]. However, whether the expression of vimentin is also regulated by PPAR*α* is still not clear. In this study, we investigated the role of Smad2/3 in regulating vimentin mRNA expression and the relationship of Smad2/3 and PPAR*α* in vimentin distribution in SCs.

Our qRT-PCR results showed that vimentin mRNA expression was upregulated in DBP-exposed SCs at 24 h. A previous microarray study showed that vimentin was upregulated in DBP-treated rat testes at gestational day 20 (GEO accession number: GSE13550). A previous proteomics study also showed that vimentin was upregulated in DBP-exposed rat testes [13]. These results indicated that vimentin expression was stimulated by DBP. Previous studies have indicated that Smad2/3 regulates the expression of vimentin, and the targets were bound by p-Smad2/3 at SBE-specific sequence GTCTG or CAGAC [30, 31]. In this study, we found an SBE sequence in the promoter of vimentin which was located at −837 to −924 bp relative to the transcriptional start site by ChIP-qPCR. Moreover, 12 bp downstream of our selected target region for the ChIP-qPCR assay is an AP1 target site. The AP1 site may facilitate the binding of p-Smad2/3 to its target sequence [17]. We hypothesized that Smad2/3 interacts with other transcriptional factors in regulating the expression of vimentin.

Next, we investigated the influence of Smad2/3 on the phosphorylation and solubility of vimentin. The results (as shown in Figures 2(b), 2(c), 2(e), and 2(f)) demonstrated that the inhibition of Smad2/3 had an inhibitory effect on the phosphorylation of vimentin compared with DBP-treated SCs. The effect of Smad2/3 on the solubility of vimentin was apparent as the relative concentration of soluble vimentin decreased significantly (*P* < 0.05) in a time-dependent manner in SB431542 + DBP-treated SCs. This was consistent with previous studies [14, 15, 32, 33]. In these studies, the upregulation of vimentin was closely related to the stimulation of Smad2/3, and SB431542 treatment directly inhibited the expression of vimentin. These results suggested that the regulation of vimentin expression by Smad2/3 may be a universal mechanism.

To illuminate the relationship between PPAR*α* and Smad2/3, GW6471, WY14643, and SB431542 were used. The results showed that the activity of Smad2/3 was promoted by WY14643 and inhibited by GW6471. The relationship between PPAR*α* and Smad2/3 is similar to a previous study, where TGF*β*1-induced *α*-SMA was elevated in the WY14643 pretreated group, while it was inhibited in the GW6471 pretreated group [34]. Another study showed that WY14643 could upregulate the mRNA expression of TGF*β* in lung allografts and spleens [35]. Additionally, SB431542 was used to inhibit the activity of p-Smad2/3, and the results showed that activity of PPAR*α* was not affected by SB431542 at 1 h but decreased after 3 h. These results suggested that PPAR*α* stimulates the activation of p-Smad2/3 in DBP-exposed SCs, and Smad2/3 may initiate a positive feedback loop with PPAR*α*.

The disrupted vimentin cytoskeleton in DBP-treated SCs was not recovered by SB431542 but showed a new abnormal distribution, mainly consisting of abundant irregular long filamentous structures. Phosphorylation of vimentin in SB431542 + DBP-treated SCs was inhibited compared to the DBP-treated group. SB431542 inhibited the expression of vimentin. Abnormally high and low levels of phosphorylation both affected the dynamic reassembly of vimentin [36]. These two factors may be reasons for the abnormal distribution of vimentin in SB431542 + DBP-treated SCs. Studying the interaction of Smad2/3 with other kinases which affected the posttranslational modifications of vimentin in DBP-treated SCs would be our next objective. The mechanism behind the irregular long filamentous structures of vimentin may involve signaling transduction along these filaments [37].

We determined that abnormal stimulation of the activity of Smad2/3 in SCs by DBP disrupted the dynamic assembly of connections between SCs and the developing spermatids, and the effect of Smad2/3 on vimentin was one aspect of a cascade of responses induced by DBP. Proliferation of SCs is mediated by activin through Smad3; Smad3 knockout mice exhibited delayed SCs maturation [38, 39]. Sertoli epithelium separates germ cells from testis interstitium and cooperates with peritubular myoid cells to facilitate the spermatogenesis [40]. DBP, as a phthalate, could induce Leydig cells hyperplasia and then deprive the space belonging to SCs [41]. According to a recent study, SCs could be reprogrammed to Leydig cells by Wt1 ablation [42]. Two previous gene array studies showed that Wt1 was downregulated in testes induced by DBP (GSE25196, GSE13550). Upregulation of Vimentin, as a marker of epithelial-to-mesenchyme transformation [43–45], by Smad2/3 induced by DBP in SCs may also trigger the epithelial-to-mesenchyme transformation, disturb the epithelial layer, and affect the stability of blood testes barrier. Disruption of blood testes barrier is one of the major adverse effects induced by DBP [46]. Besides, cross talk of TGF*β*1/Smad3 with orphan nuclear receptor Nur77 was reported to repress the steroidogenesis in Leydig cells [47]. Vimentin participates in the transportation of cholesterol during steroidogenesis [48–50]. Steroidogenesis is disrupted by DBP according to several previous studies [51, 52]. PPAR*α* was reported to be an indirect transrepressor of SF1 on steroidogenic genes in testes [53]. To some extent, PPAR*α* may also affect the steroidogenesis by affecting vimentin in DBP-exposed testes cells. Although we performed our experiment in SCs here, there is vimentin in Leydig cells too [54]. So mechanism deduced here may also shed some light on DBP-exposed Leydig cells studies.

A limitation of this research is that we only studied the expression and phosphorylation of vimentin in DBP-exposed SCs. In physiological situations, ubiquitination, glycosylation, acetylation, and many other modifications affect the distribution and functions of vimentin [55]. In future studies, other modifications should be taken into consideration. In vivo studies are also needed to confirm the conclusions deduced from these in vitro studies.

## 5. Conclusion

**Figure 5:**
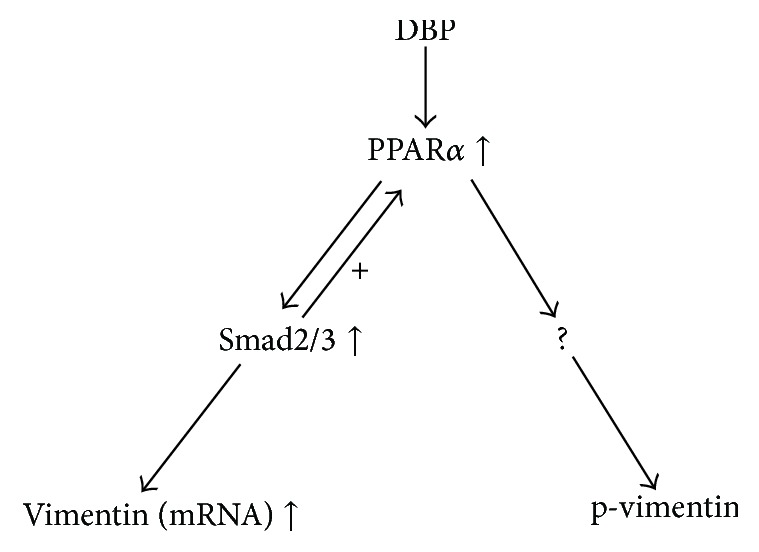
Schematic diagram of the relationship between PPAR*α* and Smad2/3 in regulation of vimentin in DBP-exposed SCs. DBP stimulated the activity of PPAR*α*, activated PPAR*α* promoted the activity of Smad2/3, and activated Smad2/3 upregulated the vimentin mRNA expression. As SB431542 inhibited Smad2/3 and could inhibit PPAR*α* to some extent, we deduced that Smad2/3 may have a positive feedback response to PPAR*α*.

In this study, we found that Smad2/3 upregulated the expression of vimentin in DBP-exposed SCs. The inhibition of PPAR*α* could attenuate the activity of Smad2/3. Smad2/3 may have a positive feedback effect on PPAR*α*. Smad2/3 and PPAR*α* cooperated in regulating the expression, phosphorylation, and distribution of vimentin in DBP-exposed SCs (as shown in the schematic diagram in Figure 5).

## Figures and Tables

**Table 1:** List of primers for real-time PCR and ChIP-qPCR.

Primer name	Sequences (5′ to 3′)	*T*_*m*_ (°C)	Target	Product size (bp)
vimentin_LEFT	GCACCCTGCAGTCATTCAGA	59	+618 to +699	81
vimentin_RIGHT	GCAAGGATTCCACTTTACGTTCA	61.2		

actin_LEFT	CCCTGGCTCCTAGCACCAT	60	+982 to +1061	79
actin_RIGHT	AGAGCCACCAATCCACACAGA	60		

SBE1_LEFT	TTTCATGACGTTTCTTTGTGG	54.1	−1356 to −1442	87
SBE1_RIGHT	TTAGAGCAGCGATGCAGG	57.3		

SBE2_LEFT	GGCGGCAGGGAGACTTAG	61.9	−1018 to −1101	84
SBE2_RIGHT	CGGGGGAGTCACAAAACA	57.3		

SBE3_LEFT	TCTGGGAGGAATGGGATG	57.3	−972 to −1053	82
SBE3_RIGHT	TGTGGGAGTGTGGAGGGT	59.6		

SBE4_LEFT	AGCGAGTTCAGCGCTCTT	57.3	−837 to −924	88
SBE4_RIGHT	CATGGATCCCTGAGGGTG	59.6		
